# 

**Published:** 1861-10

**Authors:** 


### CONTENTS:

### ORIGINAL COMMUNICATIONS.

PAGE.
ART. I.—Letter from Williamsburg. By Dr. J. G. Westmoreland... 63

### SELECTIONS.

Opium.......................................................... 69
Urine .......................................................... 76
Uterine Hemoorhage.............................................. 95
Placental Complications......................................... 103

### EDITORIAL AND MISCELLANEOUS.

Suspension...................................................... 111

Atlanta Medical College......................................... 112

Annual Announcement of Lectures in the Atlanta Medical College.. 112

